# Supplementary material for: A Likelihood Approach for Real-Time Calibration of Stochastic Compartmental Epidemic Models
Source: PLoS Comput Biol. 2017 Jan 17;13(1):e1005257. doi: 10.1371/journal.pcbi.1005257 (PMC5240920; doi:10.1371/journal.pcbi.1005257)
Supplement: S1 File — (TAR.GZ) [file pcbi.1005257.s014.tar.gz › HSPH_Online-SI-Revision/Additions/PF09/n100-extreme-SEITR/n100-extreme-SEITR_PF_table4.pdf]

1	R0	{1.38654, 9.85565}	{2.20019, 20.}	{{2.19568, 20.}, {2.20341, 20.}}	{58.6819, 102.929}	inf	602.	15 694.9	{15 612.5, 15 744.2}	229.863	0.518331	newly	2747.	5231.59	{5146., 5301.}	90.4474	0.279742	Reff	0.128602	0.751094	{0.749017, 0.754086}	484.048	ar	92 379.	92 039.5	{91 662., 92 321.}	0.377813
2	R0	{2.36629, 12.2029}	{3., 20.}	{{3., 20.}, {3., 20.}}	{26.7807, 63.8955}	inf	104.	22 920.2	{22 873.5, 22 954.9}	289.005	0.589955	newly	2664.	7207.7	{7117., 7288.}	170.559	0.432242	Reff	0.0301229	0.409607	{0.408399, 0.411094}	1259.79	ar	99 022.	101 343.	{101 033., 101 639.}	2.34343
3	R0	{2.32253, 12.836}	{3., 20.}	{{3., 20.}, {3., 20.}}	{29.1695, 55.8118}	inf	185.	23 894.1	{23 839., 23 952.8}	205.395	0.484861	newly	3413.	7556.07	{7442., 7645.}	121.391	0.34514	Reff	0.035767	0.449337	{0.447362, 0.450947}	1156.29	ar	98 997.	100 845.	{100 396., 101 180.}	1.86711
4	R0	{1.79075, 11.2058}	{3., 20.}	{{3., 20.}, {3., 20.}}	{67.5276, 78.479}	inf	424.	20 272.2	{20 073.4, 20 381.7}	201.984	0.479977	newly	3304.	6332.06	{6228., 6423.}	91.6483	0.282476	Reff	0.0739401	0.354083	{0.350781, 0.35748}	378.878	ar	96 920.	103 914.	{103 313., 104 778.}	7.21621
5	R0	{2.26952, 9.62371}	{3., 20.}	{{3., 20.}, {3., 20.}}	{32.1865, 107.82}	inf	39.	24 585.6	{24 522.2, 24 641.}	891.353	0.996228	newly	1318.	7787.25	{7678., 7895.}	490.838	0.771444	Reff	0.0289591	0.456056	{0.454216, 0.458068}	1474.83	ar	98 881.	101 799.	{101 423., 102 121.}	2.95095
6	R0	{1.57191, 6.89004}	{3., 20.}	{{3., 20.}, {3., 20.}}	{90.8506, 190.274}	inf	100.	22 000.	{21 894.7, 22 102.1}	1416.19	1.18075	newly	1057.	6893.79	{6804., 6986.}	552.203	0.814361	Reff	0.0825567	0.385179	{0.382655, 0.387906}	366.563	ar	95 046.	104 162.	{103 652., 104 596.}	9.59125
7	R0	{1.99226, 17.2339}	{2.12463, 20.}	{{2.12276, 20.}, {2.12731, 20.}}	{6.6441, 16.0503}	inf	602.	16 380.7	{16 329.9, 16 417.}	42.7387	0.15454	newly	3977.	5446.65	{5361., 5522.}	36.9537	0.136545	Reff	0.0686533	0.722855	{0.721048, 0.723866}	952.907	ar	97 992.	90 046.4	{89 769., 90 354.}	8.10838
8	R0	{2.63369, 15.1879}	{3., 20.}	{{3., 20.}, {3., 20.}}	{13.9086, 31.6838}	inf	100.	21 462.2	{21 424.8, 21 491.4}	161.639	0.417702	newly	3068.	6724.8	{6641., 6819.}	119.192	0.340799	Reff	0.022123	0.387994	{0.386915, 0.38904}	1653.81	ar	99 451.	101 308.	{100 989., 101 522.}	1.86736
9	R0	{2.53271, 9.90552}	{3., 20.}	{{3., 20.}, {3., 20.}}	{18.4502, 101.908}	inf	27.	27 579.6	{27 491.4, 27 669.2}	969.805	1.0293	newly	1330.	8863.55	{8775., 8965.}	566.432	0.823741	Reff	0.0191979	0.555905	{0.553251, 0.55881}	2795.65	ar	99 355.	101 548.	{101 225., 101 854.}	2.20754
10	R0	{2.48791, 18.9003}	{2.79946, 20.}	{{2.78346, 20.}, {2.8464, 20.}}	{12.5226, 5.81843}	inf	387.	19 844.3	{19 793.7, 19 867.6}	32.4191	0.12195	newly	4707.	6295.83	{6195., 6384.}	33.7546	0.126281	Reff	0.0381148	0.454656	{0.44582, 0.457946}	1092.86	ar	99 291.	97 559.4	{97 047., 98 885.}	1.74398
11	R0	{2.84414, 18.4765}	{2.26655, 20.}	{{2.2598, 20.}, {2.26968, 20.}}	{20.308, 8.24561}	inf	161.	21 081.1	{20 996.3, 21 144.5}	57.8871	0.198345	newly	4255.	7212.96	{7128., 7317.}	69.5173	0.229193	Reff	0.0214164	0.91938	{0.916911, 0.923626}	4192.89	ar	99 654.	92 881.4	{92 563., 93 162.}	6.7961
12	R0	{2.29369, 14.622}	{2.92013, 20.}	{{2.91577, 20.}, {2.92357, 20.}}	{27.3115, 36.7802}	inf	189.	19 602.4	{19 588.4, 19 612.9}	148.32	0.395012	newly	3139.	6183.52	{6100., 6272.}	96.9901	0.294418	Reff	0.0347723	0.420172	{0.419012, 0.421512}	1108.35	ar	98 982.	98 766.1	{98 629., 98 894.}	0.220697
13	R0	{2.73413, 16.7529}	{2.80461, 20.}	{{2.80302, 20.}, {2.80654, 20.}}	{2.57769, 19.3823}	inf	128.	22 278.	{22 266.3, 22 288.5}	116.648	0.335755	newly	3594.	7129.36	{7026., 7222.}	98.3684	0.29745	Reff	0.0212989	0.516077	{0.514815, 0.517007}	2323.03	ar	99 543.	97 206.6	{97 056., 97 369.}	2.34713
14	R0	{1.79554, 16.5943}	{1.9393, 20.}	{{1.93624, 20.}, {1.94211, 20.}}	{8.0067, 20.5233}	inf	1182.	14 885.4	{14 839.5, 14 933.1}	9.99322	0.0413648	newly	4850.	5140.14	{5072., 5209.}	5.98227	0.0252088	Reff	0.106063	0.9446	{0.942937, 0.946123}	790.606	ar	96 821.	83 536.3	{83 221., 83 841.}	13.7208
15	R0	{1.91429, 13.7771}	{1.92017, 20.}	{{1.91864, 20.}, {1.92281, 20.}}	{0.364437, 45.1684}	inf	383.	14 390.8	{14 369.8, 14 439.2}	77.8394	0.250018	newly	3380.	5076.63	{4978., 5151.}	50.1962	0.176611	Reff	0.0667896	1.0692	{1.06812, 1.07011}	1500.85	ar	97 541.	82 642.9	{82 330., 82 892.}	15.2737
16	R0	{1.42094, 10.5871}	{2.16033, 17.2247}	{{2.15787, 17.1946}, {2.16245, 17.26}}	{52.0351, 62.6956}	inf	1054.	15 878.6	{15 842.3, 15 917.2}	113.825	0.330058	newly	4015.	6010.63	{5930., 6096.}	49.7044	0.17521	Reff	0.134932	0.682512	{0.680677, 0.684479}	405.817	ar	93 044.	87 854.9	{87 626., 88 127.}	5.57709
17	R0	{1.45515, 6.16466}	{3., 20.}	{{3., 20.}, {3., 20.}}	{106.164, 224.43}	inf	170.	24 930.8	{24 819.8, 25 001.1}	1336.93	1.15743	newly	1376.	7882.93	{7772., 8005.}	472.887	0.758042	Reff	0.105091	0.444795	{0.442998, 0.447242}	323.248	ar	93 254.	103 914.	{103 501., 104 320.}	11.431
18	R0	{2.85669, 13.2416}	{3., 20.}	{{3., 20.}, {3., 20.}}	{5.01665, 51.0391}	inf	31.	24 589.8	{24 533., 24 634.}	300.942	0.603081	newly	2583.	7805.97	{7709., 7902.}	202.206	0.480285	Reff	0.014512	0.469354	{0.467766, 0.471428}	3134.25	ar	99 644.	101 757.	{101 391., 102 015.}	2.12016
19	R0	{2.56417, 17.9558}	{2.24002, 20.}	{{2.23867, 20.}, {2.24115, 20.}}	{12.6415, 11.3846}	inf	266.	20 355.7	{20 331.1, 20 377.3}	47.2705	0.168116	newly	4524.	6899.31	{6796., 6993.}	52.5046	0.183262	Reff	0.0335137	0.866833	{0.865741, 0.868145}	2486.5	ar	99 389.	91 674.5	{91 435., 91 935.}	7.76198
20	R0	{2.27517, 11.1864}	{3., 20.}	{{3., 20.}, {3., 20.}}	{31.8583, 78.7885}	inf	92.	22 276.2	{22 177.6, 22 357.8}	414.699	0.711551	newly	2013.	6988.	{6891., 7073.}	247.144	0.540486	Reff	0.0313746	0.389447	{0.387376, 0.391843}	1141.28	ar	98 894.	104 344.	{103 812., 104 806.}	5.5108
21	R0	{2.35735, 10.4735}	{3., 20.}	{{3., 20.}, {3., 20.}}	{27.2615, 90.9581}	inf	65.	25 395.6	{25 291.9, 25 456.9}	582.677	0.834214	newly	1848.	8046.59	{7936., 8132.}	335.422	0.638891	Reff	0.0275103	0.467514	{0.465811, 0.469421}	1599.41	ar	99 036.	102 711.	{102 171., 103 076.}	3.71085
22	R0	{1.79551, 11.9179}	{2.99967, 20.}	{{3., 20.}, {3., 20.}}	{67.0648, 67.8148}	inf	355.	18 145.	{18 015.8, 18 234.6}	191.392	0.464471	newly	2882.	5667.54	{5564., 5762.}	96.653	0.293661	Reff	0.0710663	0.352589	{0.350231, 0.355431}	396.14	ar	97 033.	100 779.	{100 169., 101 551.}	3.86104
23	R0	{1.23545, 6.31687}	{3., 20.}	{{3., 20.}, {3., 20.}}	{142.827, 216.612}	inf	264.	16 999.5	{16 971.2, 17 024.8}	924.685	1.01059	newly	1370.	5279.47	{5185., 5349.}	285.363	0.585838	Reff	0.148353	0.323319	{0.322032, 0.324728}	117.939	ar	88 739.	99 931.	{99 672., 100 152.}	12.6122
24	R0	{2.24089, 10.1789}	{3., 20.}	{{3., 20.}, {3., 20.}}	{33.8754, 96.4849}	inf	88.	26 368.5	{26 320., 26 410.5}	529.019	0.798663	newly	2182.	8417.41	{8324., 8507.}	285.766	0.586306	Reff	0.0325825	0.511196	{0.509849, 0.512848}	1468.93	ar	98 868.	100 893.	{100 574., 101 157.}	2.04837
25	R0	{1.57298, 10.2607}	{2.17206, 16.6185}	{{2.16637, 16.59}, {2.17421, 16.6585}}	{38.0857, 61.963}	inf	633.	17 042.7	{17 013.5, 17 078.7}	159.836	0.414699	newly	3489.	6750.95	{6655., 6840.}	93.4924	0.286636	Reff	0.10572	0.741608	{0.738902, 0.745488}	601.483	ar	94 957.	87 408.9	{87 180., 87 660.}	7.949
26	R0	{1.2154, 8.2576}	{2.15699, 16.9792}	{{2.15514, 16.9426}, {2.1588, 17.0102}}	{77.4716, 105.619}	inf	684.	14 151.1	{14 111.8, 14 204.3}	265.19	0.562517	newly	2648.	5363.56	{5288., 5442.}	102.551	0.306508	Reff	0.165416	0.60883	{0.606754, 0.610728}	268.06	ar	88 421.	88 892.1	{88 687., 89 085.}	0.532792
27	R0	{2.22841, 18.7983}	{2.17613, 17.039}	{{2.17352, 16.9836}, {2.17831, 17.093}}	{2.34625, 9.35873}	inf	454.	15 107.5	{15 027.1, 15 182.9}	16.7415	0.0672213	newly	4125.	5715.17	{5620., 5790.}	38.5496	0.141576	Reff	0.0518997	0.612015	{0.609147, 0.615118}	1079.23	ar	98 756.	91 746.3	{91 485., 92 020.}	7.09799
28	R0	{1.27778, 5.36767}	{3., 20.}	{{3., 20.}, {3., 20.}}	{134.782, 272.601}	inf	160.	22 782.8	{22 732.7, 22 824.3}	1946.97	1.31111	newly	965.	7179.41	{7094., 7265.}	643.98	0.871541	Reff	0.136275	0.415013	{0.413625, 0.416905}	204.54	ar	89 843.	101 567.	{101 199., 101 873.}	13.0492
29	R0	{2.38042, 15.8901}	{3., 20.}	{{3., 20.}, {3., 20.}}	{26.0282, 25.8645}	inf	266.	21 146.	{21 114.1, 21 176.5}	80.2883	0.255967	newly	4278.	6665.84	{6587., 6763.}	55.8167	0.192591	Reff	0.0399673	0.408547	{0.407357, 0.409897}	922.205	ar	99 112.	97 947.8	{97 808., 98 088.}	1.17461
30	R0	{2.53188, 12.7607}	{3., 20.}	{{3., 20.}, {3., 20.}}	{18.489, 56.7312}	inf	140.	26 760.8	{26 661.3, 26 828.1}	230.462	0.519121	newly	3519.	8534.48	{8438., 8628.}	142.526	0.384737	Reff	0.0262556	0.504069	{0.502194, 0.506205}	1819.85	ar	99 317.	102 987.	{102 553., 103 432.}	3.69568
31	R0	{1.24482, 10.5471}	{2.28836, 20.}	{{2.28676, 20.}, {2.2893, 20.}}	{83.8305, 89.6256}	inf	1178.	14 396.8	{14 371.5, 14 418.1}	140.107	0.380404	newly	3288.	4621.51	{4544., 4708.}	40.5569	0.147811	Reff	0.174511	0.511844	{0.510527, 0.513374}	193.301	ar	89 097.	96 385.3	{96 084., 96 679.}	8.18021
32	R0	{2.30446, 17.9607}	{3., 20.}	{{3., 20.}, {3., 20.}}	{30.1823, 11.3542}	inf	386.	17 299.6	{17 255.6, 17 365.1}	30.6323	0.116049	newly	4308.	5379.57	{5295., 5455.}	24.874	0.0964432	Reff	0.0453748	0.337302	{0.335358, 0.339575}	643.368	ar	99 028.	98 444.9	{98 204., 98 631.}	0.588773
33	R0	{2.31579, 11.8236}	{3., 20.}	{{3., 20.}, {3., 20.}}	{29.5454, 69.1532}	inf	79.	21 096.5	{21 013.3, 21 167.}	380.667	0.681842	newly	2012.	6579.96	{6475., 6670.}	227.036	0.514568	Reff	0.0280905	0.359482	{0.357041, 0.362264}	1179.73	ar	99 012.	105 236.	{104 658., 105 634.}	6.28568
34	R0	{1.6678, 10.6744}	{3., 20.}	{{3., 20.}, {3., 20.}}	{79.8777, 87.3642}	inf	325.	16 290.3	{16 250.7, 16 319.4}	247.787	0.541312	newly	2446.	5064.89	{4967., 5131.}	107.068	0.316082	Reff	0.0850078	0.321451	{0.320459, 0.322799}	278.144	ar	95 809.	99 003.1	{98 850., 99 153.}	3.33377
35	R0	{1.65476, 7.07559}	{3., 20.}	{{3., 20.}, {3., 20.}}	{81.2952, 182.662}	inf	106.	24 922.1	{24 835.4, 24 978.6}	1377.3	1.16947	newly	1151.	7900.39	{7807., 8001.}	586.394	0.836553	Reff	0.0754074	0.460903	{0.459142, 0.462727}	511.217	ar	95 746.	102 747.	{102 335., 103 212.}	7.31189
36	R0	{2.71807, 18.5959}	{2.94816, 20.}	{{2.93889, 20.}, {2.95365, 20.}}	{8.46507, 7.55059}	inf	279.	22 040.3	{22 001.5, 22 072.3}	41.4111	0.150483	newly	5029.	6967.67	{6860., 7073.}	38.5498	0.141576	Reff	0.0291921	0.432669	{0.430333, 0.435992}	1382.14	ar	99 540.	99 885.5	{99 630., 100 110.}	0.361111
37	R0	{2.6202, 12.7552}	{2.8645, 20.}	{{2.85825, 20.}, {2.86743, 20.}}	{9.32369, 56.7988}	inf	74.	26 429.3	{26 377.1, 26 459.9}	294.703	0.59627	newly	2840.	8596.01	{8474., 8722.}	202.676	0.480956	Reff	0.0220097	0.625357	{0.623167, 0.628952}	2741.28	ar	99 426.	99 065.8	{98 789., 99 289.}	0.3623
38	R0	{1.47672, 9.81651}	{2.13912, 16.551}	{{2.13707, 16.5507}, {2.13952, 16.5975}}	{44.8564, 68.604}	inf	503.	15 160.9	{15 136.6, 15 202.7}	225.132	0.512057	newly	2643.	5945.54	{5876., 6027.}	124.954	0.352071	Reff	0.109425	0.670661	{0.669701, 0.672949}	512.895	ar	93 815.	87 274.8	{87 022., 87 490.}	6.97137
39	R0	{1.90004, 12.3313}	{2.87608, 20.}	{{2.87265, 20.}, {2.87861, 20.}}	{51.3693, 62.1889}	inf	369.	19 271.4	{19 248.6, 19 290.6}	164.064	0.421709	newly	3317.	6085.9	{5990., 6165.}	83.476	0.263551	Reff	0.0681354	0.423648	{0.422332, 0.425177}	521.774	ar	97 413.	99 064.7	{98 895., 99 215.}	1.69561
40	R0	{2.64218, 18.5237}	{3., 20.}	{{3., 20.}, {3., 20.}}	{13.5426, 7.96979}	inf	174.	16 972.	{16 924.2, 17 003.6}	45.9454	0.16419	newly	3674.	5283.25	{5198., 5358.}	43.801	0.157737	Reff	0.0267917	0.33562	{0.334565, 0.336758}	1152.7	ar	99 474.	100 391.	{100 124., 100 574.}	0.921688
41	R0	{1.6945, 7.17996}	{3., 20.}	{{3., 20.}, {3., 20.}}	{77.0434, 178.553}	inf	141.	26 489.4	{26 416.6, 26 533.8}	963.831	1.02687	newly	1635.	8484.82	{8389., 8580.}	418.949	0.715107	Reff	0.0694067	0.527418	{0.526341, 0.528635}	659.895	ar	96 332.	101 331.	{101 089., 101 557.}	5.18899
42	R0	{1.84559, 18.0074}	{2.28211, 20.}	{{2.27291, 20.}, {2.28972, 20.}}	{23.6521, 11.0655}	inf	1048.	16 316.3	{16 261.2, 16 361.6}	19.2796	0.076565	newly	4650.	5279.74	{5183., 5363.}	13.5428	0.055126	Reff	0.093313	0.573863	{0.570117, 0.576852}	514.987	ar	97 358.	96 328.1	{95 664., 96 864.}	1.0579
43	R0	{2.24965, 17.0361}	{2.88368, 20.}	{{2.87855, 20.}, {2.88866, 20.}}	{28.1835, 17.3978}	inf	520.	19 355.	{19 307., 19 441.1}	43.9032	0.158069	newly	4598.	6112.99	{6020., 6204.}	32.9489	0.123654	Reff	0.0537891	0.424238	{0.422536, 0.425801}	688.705	ar	98 770.	99 316.5	{99 150., 99 468.}	0.553336
44	R0	{2.54324, 15.7967}	{2.99993, 20.}	{{3., 20.}, {3., 20.}}	{17.957, 26.6087}	inf	127.	19 902.1	{19 860.4, 19 933.}	124.249	0.35073	newly	3246.	6237.64	{6157., 6324.}	92.1639	0.283645	Reff	0.0255087	0.376628	{0.375658, 0.378295}	1376.47	ar	99 354.	99 837.7	{99 550., 100 099.}	0.486805
45	R0	{2.54943, 14.8032}	{3., 20.}	{{3., 20.}, {3., 20.}}	{17.6734, 35.1059}	inf	120.	21 187.	{21 135.8, 21 227.4}	159.741	0.414539	newly	3187.	6632.12	{6555., 6702.}	108.099	0.318254	Reff	0.0250609	0.382926	{0.38175, 0.384187}	1427.98	ar	99 380.	101 083.	{100 748., 101 393.}	1.71315
46	R0	{2.37755, 12.6461}	{3., 20.}	{{3., 20.}, {3., 20.}}	{26.1803, 58.1515}	inf	129.	23 782.5	{23 729.9, 23 822.1}	228.488	0.516519	newly	3124.	7540.64	{7460., 7627.}	141.378	0.382679	Reff	0.0309795	0.445345	{0.443905, 0.447223}	1337.55	ar	99 096.	100 508.	{100 215., 100 788.}	1.42479
47	R0	{1.69279, 11.6636}	{2.23605, 17.5615}	{{2.22549, 17.5376}, {2.24332, 17.592}}	{32.0926, 50.5663}	inf	535.	18 485.6	{18 398.7, 18 553.5}	147.267	0.393163	newly	3657.	6954.8	{6861., 7051.}	90.1777	0.279136	Reff	0.0899041	0.752246	{0.74798, 0.758125}	736.721	ar	96 214.	90 015.6	{89 773., 90 226.}	6.44231
48	R0	{1.82402, 10.0038}	{3., 20.}	{{3., 20.}, {3., 20.}}	{64.4719, 99.924}	inf	312.	22 615.8	{22 568., 22 651.6}	332.59	0.636076	newly	2763.	7115.38	{7008., 7203.}	157.524	0.410787	Reff	0.0676164	0.406344	{0.404724, 0.407978}	500.954	ar	97 076.	101 862.	{101 577., 102 128.}	4.93016
49	R0	{1.6424, 11.1667}	{3., 20.}	{{3., 20.}, {3., 20.}}	{82.6595, 79.1039}	inf	368.	14 931.9	{14 885.3, 14 975.4}	210.564	0.492145	newly	2454.	4622.14	{4533., 4704.}	88.3513	0.274928	Reff	0.0891166	0.293232	{0.291439, 0.295041}	229.043	ar	95 546.	100 371.	{100 057., 100 639.}	5.04989
50	R0	{2.05638, 14.3048}	{3., 20.}	{{3., 20.}, {3., 20.}}	{45.8874, 39.8132}	inf	362.	18 174.4	{18 115., 18 257.2}	96.3526	0.293034	newly	3752.	5669.06	{5596., 5745.}	51.0943	0.179225	Reff	0.0564271	0.337715	{0.335724, 0.340254}	498.498	ar	98 194.	100 610.	{100 308., 100 875.}	2.46087
